# Clinicians’ Perceptions and Potential Applications of Robotics for Task Automation in Critical Care: Qualitative Study

**DOI:** 10.2196/62957

**Published:** 2025-03-28

**Authors:** Jiafeng Song, Rishika Iytha Sridhar, Darlene Marie Rogers, Cheryl Hiddleson, Carolyn Davis, Tina Lynn Holden, Shanna Ramsey-Haynes, Lisa Reif, Julie Swann, Craig S Jabaley, Mary Gullatte, Rishikesan Kamaleswaran

**Affiliations:** 1 Department of Biomedical Engineering Duke University Durham, NC United States; 2 Department of Biomedical Engineering Georgia Institute of Technology Atlanta, GA United States; 3 Department of Biomedical Informatics Emory University Atlanta, GA United States; 4 Emory Hillandale Hospital Lithonia, GA United States; 5 eICU Operations Emory Healthcare Atlanta, GA United States; 6 Department of Surgery Emory University School of Medicine Atlanta, GA United States; 7 Emory Critical Care Center Emory Healthcare Atlanta, GA United States; 8 Emory University Hospital Midtown Atlanta, GA United States; 9 Emory University Hospital Atlanta, GA United States; 10 Emory Saint Joseph’s Hospital Atlanta, GA United States; 11 Department of Anesthesiology Emory University Atlanta, GA United States; 12 Nell Hodgson Woodruff School of Nursing Emory University Atlanta, GA United States; 13 Department of Anesthesiology Duke University Durham, NC United States; 14 Department of Electrical and Computer Engineering Duke University Durham, NC United States; 15 Department of Surgery Duke University School of Medicine Durham, NC United States

**Keywords:** robotics, intensive care units, critical care, health care technology, qualitative study

## Abstract

**Background:**

Interest in integrating robotics within intensive care units (ICUs) has been propelled by technological advancements, workforce challenges, and heightened clinical demands, including during the COVID-19 pandemic. The integration of robotics in ICUs could potentially enhance patient care and operational efficiency amid existing challenges faced by health care professionals, including high workload and decision-making complexities.

**Objective:**

This qualitative study aimed to explore ICU clinicians’ perceptions of robotic technology and to identify the types of tasks that might benefit from robotic assistance. We focused on the degree of acceptance, perceived challenges, and potential applications for improving patient care in 5 Southeastern US hospitals between January and August 2023.

**Methods:**

A qualitative study through semistructured interviews and questionnaires was conducted with 15 ICU clinicians (7 nurses, 6 physicians, and 2 advanced practice providers) from 5 hospitals in the Southeast United States. Directed content analysis was used to categorize and interpret participants’ statements, with statistical tests used to examine any role-based differences in how they viewed robotic integration.

**Results:**

Among the 15 participants, 73% (11/15) were female, with an average of 6.4 (SD 6.3) years of ICU experience. We identified 78 distinct tasks potentially suitable for robotic assistance, of which 50 (64%) involved direct patient care (eg, repositioning patients and assisting with simple procedures), 19 (24%) concerned indirect patient care (eg, delivering supplies and cleaning), 6 (8%) addressed administrative tasks (eg, answering call lights), and 3 (4%) were classified as mixed direct and indirect (eg, sitting with a patient to keep them calm). Most participants supported the automation of routine, noncritical tasks (eg, responding to nurse calls and measuring glucose levels), viewing this strategy as a way to alleviate workload and enhance efficiency. Conversely, high-complexity tasks requiring nuanced clinical judgment (eg, ventilator settings) were deemed unsuitable for full automation. Statistical analysis revealed no significant difference in how nurses, physicians, and advanced practice providers perceived these tasks (*P*=.22).

**Conclusions:**

Our findings indicate a significant opportunity to use robotic systems to perform noncomplex tasks in ICUs, thereby potentially improving efficiency and reducing staff burden. Clinicians largely view robots as supportive tools rather than substitutes for human expertise. However, concerns persist regarding privacy, patient safety, and the loss of human touch, particularly for tasks requiring high-level clinical decision-making. Future research should involve broader, more diverse clinician samples and investigate the long-term impact of robotic assistance on patient outcomes while also incorporating patient perspectives to ensure ethical, patient-centered adoption of robotic technology.

## Introduction

The intensive care unit (ICU) cohorts patients who are critically ill and who require sophisticated care to manage severe, life-threatening conditions. ICUs are both labor-intensive and technophilic, offering an opportunity to evaluate the potential role of robotics to facilitate critical care despite potential technical challenges [1]. The ICU environment is characterized by an assortment of medical devices, including ventilators, physiologic monitors, intravenous pumps and lines, feeding tubes, various drainage and catheter systems, and dialysis equipment [2]. Each device requires meticulous management, and the urgency and complexity of critical care mean that timely responses can be a matter of life and death. Health care professionals in these settings face immense challenges, including making critical decisions under pressure, caring for patients who are nonresponsive, managing equipment, and providing essential emotional support during patients’ most vulnerable moments. Furthermore, the increasing demand for critical care services exacerbates the strain on the limited pool of skilled health care workers, who operate under significant stress [3]. Despite these hurdles, the dedication and efforts of these professionals in overcoming such challenges are noteworthy.

In the health care industry, and particularly in the ICU, the impact of technological advancement has been significant. The adoption of electronic health records, for example, has streamlined access to patient histories and clinical data, facilitating more timely and more effective treatment decisions [4-7]. Given that critical care clinicians work around the clock, innovations that offload common tasks or enhance patient safety have been instrumental, such as automated intravenous pumps [8]. These devices can integrate with the electronic health record to reduce medication errors, ensure the precise delivery of medications, and automatically document medication administration, significantly alleviating the workload of nursing staff while minimizing the potential for errors.

Robotic technology in ICUs, although not widespread, shows potential for enhancing patient care management. Telepresence robots, for example, have dramatically decreased response times to patients, facilitating timelier interventions and contributing to a reduction in mortality [1,9,10]. In the field of physical therapy and rehabilitation, particularly for survivors of stroke, rehabilitation robots have demonstrated efficacy, improved quality of care, and effectively customized rehabilitation activities for motor function recovery [11-16]. Additionally, robots dedicated to medication and equipment delivery and remote patient monitoring have streamlined care processes, enhancing efficiency in patient management [17-19]. These robotic solutions have consistently yielded positive outcomes, underscoring their value in improving patient care. However, it is crucial to acknowledge their limitations and potential concerns regarding cost-effectiveness. Despite these challenges, the contribution of robotics to health care, including ICUs, remains seemingly promising [20].

Understanding clinician perceptions of robotics within the ICU is paramount, as it directly influences the adoption, use, and effectiveness of these technologies. This study aims to explore the multifaceted views of health care professionals on the deployment of robotics in the ICU, identifying perceived benefits, challenges, and areas for improvement. By elucidating these objectives, including assessing clinician readiness, identifying potential tasks for robotic assistance, and evaluating the impact on patient care, we seek to bridge the translational gap between technological capability and clinical application at the bedside.

## Methods

### Study Design and Participants

The study was cross-sectional with a qualitative, exploratory design. Directed content analysis was used to identify prominent themes emerging from the data. Participant recruitment occurred at 2 academic medical centers with 850 and 500 staffed beds, respectively, and 3 regional or community hospitals with 100, 400, and 450 inpatient beds, respectively, within a large health care system in the Southeastern United States. Additionally, 2 of the hospitals held Magnet designation during the study period. The inclusion criteria for this study were registered nurses, doctors, physicians, and therapists working in ICUs in these hospitals, with experience in treating or caring for patients. Participants were required to be adults aged 18 to 80 years, capable of understanding the study’s purpose and risks, and able to provide informed consent. Additionally, participants needed to be available for a maximum 1-hour Zoom interview (Zoom Video Communications) and actively engaged in providing direct patient care in an ICU setting. Exclusion criteria included individuals with no experience in patient care or treatment, pregnant individuals, prisoners, and those who did not work directly in an ICU setting. These criteria were designed to ensure that participants had relevant clinical experience and could contribute meaningful insights into the study objectives.

### Study End Points

Our primary end point was to explore the perceptions of ICU clinicians regarding the integration of robotic technology, focusing on identifying tasks that could reduce staff workload and enhance operational efficiency. The secondary end point was to characterize the ethical and practical considerations—such as privacy, safety, and clinical judgment—surrounding the implementation of robotics in critical care environments.

### Sampling Method and Sample Size Calculation

Convenience sampling was used to recruit participants through institutional review board (IRB)–approved flyers. Given the exploratory and qualitative nature of this study, we applied a data saturation approach to inform the sample size. To align with best practices in qualitative research, we referenced the guidelines of Guest et al [21-23], which indicate that 80% to 92% of all concepts within a dataset are typically identified within the first 10 interviews, with 12 to 15 interviews generally sufficient to achieve thematic saturation. Based on this approach, we determined to enroll a minimum of 10 participants to begin the determination of data saturation.

### Recruitment Process

For recruitment, a local site contact at each hospital initially posted the flyers with the sign-up QR code on the participating units. The site contacts also introduced the study in ICU huddles and meetings and emailed the recruitment flyers to unit staff on participating units. Once potential participants expressed interest, the research team emailed them directly to confirm their availability for an interview. Follow-up communication included proposed dates and times for the interview, with flexibility to accommodate participants’ schedules. Site contacts repeated emails and huddle and meeting attendance until multiple professions were represented and data saturations were reached.

### Data Collection

Data collection began in January 2023 and concluded in August 2023 upon achieving the planned 15 interviews. Each study participant provided informed consent to be interviewed and recorded prior to the interview sessions and could withdraw at any point prior to or during the interview. For consistency, 1 research team member (JS) conducted all interviews using a structured interview guide. In addition to asking the questions, the interviewer displayed the questions in Zoom. A detailed interview script is listed in Multimedia Appendix 1.

After the interview, participants filled out a Google Forms questionnaire (Multimedia Appendix 2), which was used to collect relevant information concerning participation demographics and experience while also assessing participant perceptions using a mix of rank order, Likert scale, and yes or no questions. This included reviewing a provided list of tasks derived from existing literature (Multimedia Appendix 3) [24], with the option to suggest additional tasks not listed. All interviews were recorded and transcribed into text files via Zoom. The 3 coders (JS, RIS, and DMR) listened to the recordings and reviewed the text files to correct any obvious transcription errors (eg, “I see you” to ICU).

### Analysis

The 3 coders (JS, RIS, and DMR) applied directed content analysis to analyze the transcripts. In content analysis, coders systematically read, listen to, or view communications and assign codes to recurring patterns of communication [25]. In the first cycle of coding, the 3 coders (JS, RIS, and DMR) read each transcript independently to identify analysis categories. Initial categories were correlated to the directed questions in the interview script for descriptive, process, and value coding [26]. Each statement documented from a participant was a unit of analysis for the coders to assign to a category. If a single statement matched multiple categories such as a process and a value, it could be double-coded. Coding categories were finalized after 3 cycles, and the detailed primary codes were abstracted to secondary codes. Interviews and recruitment continued until no new secondary codes emerged. Near data saturation was observed after 11 participants, as comments began to repeat without introducing new insights. By participant 13, true data saturation was achieved, ensuring comprehensive coverage of the interview scope, which is reasonable given the narrow focus of the interview script.

To promote the trustworthiness of the analysis process, the 3 coders (JS, RIS, and DMR) debriefed after each coding cycle. The coders had different professional backgrounds: 2 (JS and RIS) were graduate students in engineering programs and 1 (DMR) was a registered nurse. Coders generated audit trail documents throughout all coding cycles and debrief sessions. All 3 coders (JS, RIS, and DMR) reviewed every unit of analysis. If there was disagreement or uncertainty among the coders about any participant statement, the coders reviewed the transcripts and relistened to the recordings until a consensus was reached. Once final coding was reached, a Python (Python Software Foundation) script was written to tally frequencies within each category.

### Statistical Plan

To examine differences in the distribution of thematic codes across participant groups, a chi-square test of independence was conducted. A contingency table was constructed using the frequency of statements categorized under each code by the 3 participant groups. The test assessed whether the observed frequencies of codes were independent of participant roles, with a significance level set at α=.05. This analysis was used to determine if there were significant differences in how each group contributed to various themes, helping to identify potential biases or group-specific priorities in the data.

### Ethical Considerations

The Emory University IRB approved this study (IRB STUDY00003902). Consent was obtained from participants both in signature form and prior to the interviews using an IRB-approved oral consent script. Interview recordings, transcripts, and participant data from the surveys were stored on a secured server in the Department of Biomedical Informatics. Participants were assigned ID codes, and transcripts were deidentified prior to analysis to keep responses anonymous. Participants will not be individually identified in any publications, presentations, or supplemental materials. Participants received a US $10 electronic Amazon gift card after each interview.

## Results

### Participants

After successfully concluding the recruitment phase, our study enlisted 15 participants, comprising 7 registered nurses, 6 physicians, and 2 advanced practice providers (APPs). The demographic profile of the study participants is summarized (Table 1). The sex distribution was predominantly female, with 11 (73%) participants, compared to 4 (27%) male participants. The average age of the participants was 35.9 (SD 8.5) years, and the ages ranged from younger physicians with an average age of 31.8 (SD 2.0) years to APPs with an average age of 42.0 (SD 5.7) years.

Experience in the ICU varied, with nurses and APPs having an average of 8.9 (SD 8.2) and 8.5 (SD 2.1) years, respectively, while physicians had a notably shorter average duration of 2.9 (SD 2.0) years. Exposure to health care robots ranged from 6 (40%) of the participants having encountered nonsurgical health care robots and 4 (27%) having experience with surgical robots.

**Table 1:** Demographic characteristics of 15 intensive care unit (ICU) clinicians from 6 hospitals in the Southeastern United States (January to August 2023), describing participant roles, age, sex, years of ICU experience, and prior exposure to robotic systems.

		Overall (N=15)	Nurse (n=7)	Physician (n=6)	APP^a^ (n=2)
**Sex, n (%)**					
	Female	11 (73)	7 (100)	3 (50)	1 (50)
	Male	4 (27)	0 (0)	3 (50)	1 (50)
Age (years), mean (SD)		35.9 (8.5)	38.0 (11.7)	31.8 (2.0)	42.0 (5.7)
Working in the ICU (years), mean (SD)		6.4 (6.3)	8.9 (8.2)	2.9 (2.0)	8.5 (2.1)
Previous encounter with a robot in health care (except for surgical robot), n (%)		6 (40)	1 (14)	4 (67)	1 (50)
Previous encounter with a surgical robot, n (%)		4 (27)	2 (29)	2 (33)	0 (0)

^a^APP: advanced practice provider.

### Coding Analysis

After the completion of the first and second cycles of coding analysis, primary codes and 7 secondary codes have been discerned. The secondary codes are administrative, comment or question about the interview, concerns, direct patient care, indirect patient care, minimum capacity, and perceptions (Table 2). Some statements were deemed not codable due to their ambiguous nature or because they were off topic.

**Table 2:** Secondary codes and associated primary codes derived from a directed content analysis of 15 intensive care unit clinicians showing the framework of perspectives on robotic integration.

Secondary codes	Primary codes
Administrative	Administrative
Comment or questions about the interview	Enquiry or comment about study
Concerns	Cost Human connection Job insecurity Lack of familiarity Legal or HIPAA^a^ Privacy—patient Privacy—clinician Quality patient care Safety—clinical judgment Safety—malfunctioning Safety—need evidence
Direct patient care	Direct—any procedure Direct—communicating Direct—food Direct—monitoring Direct—performing procedure Direct—supporting procedure Direct—transporting No physiotherapy No ventilator
Indirect patient care	Indirect—communicating Indirect—custodial Indirect—monitoring Indirect—delivering supplies Indirect—picking supplies Indirect—supporting procedure Indirect—transporting
Minimum capacity	Audio Device compatibility Dexterity Movement Simultaneous localization and mapping Storage Touch screen Video
Perceptions	“Complex solution for a simple problem” “Tasky things” Reduce burden Task delegation comfort differ by clinician User perception—fearful (patients and clinicians) User perception—need education (patients and clinicians) User perception—negative (clinicians)

^a^HIPAA: Health Insurance Portability and Accountability Act.

1. Administrative: This category pertains to the logistical and management-related facets of incorporating robots into the ICU. It addresses the administrative duties and potential ramifications therein. Statements within this category, such as “They would function akin to ICU techs and secretaries,” exemplify the envisioned roles robots could undertake to support the administrative backbone of ICU operations.
2. Comment or question about the interview: This category captures any feedback or queries participants had regarding the interview process itself, helping to clarify the interview structure or content. Some statements in this category are “What exactly is your study looking at?” or “What kind of robots you are building?”
3. Concerns: Clinicians shared a broad range of apprehensions about introducing robots into the ICU environment. Their concerns span financial aspects, workforce implications, the impact on patient privacy, the quality of health care delivery, and the loss of “human touch.”
4. Direct patient care: This category refers to tasks and activities where robots would be involved in the immediate clinical management and treatment of patients. Clinicians showed particular interest in the potential of robotics for routine tasks and procedures that require precision and can be standardized, such as turning and repositioning the patient, assisting with physical therapy, assisting bedside procedures, or checking cognition.
5. Indirect patient care: The category includes activities where robots assist with nonclinical tasks that indirectly contribute to patient well-being and the efficient functioning of the ICU. Many participants recognized the utility of robots in roles such as transporting supplies, managing inventory, and performing cleaning tasks, which, while not directly related to patient care, are vital for maintaining a safe and operational environment.
6. Minimum capacity: In addressing the potential role of robots in the ICU, our interview protocol specifically included questions aimed at understanding the essential capabilities such technologies would need. This category collects the discussions focusing on the fundamental requirements that robots must satisfy to function effectively within the intensive care framework. Participants delineated baseline functionalities that robots should possess, such as the ability to navigate complex environments, respond to dynamic situations, and interface with existing hospital systems.
7. Perceptions: Clinicians’ perceptions of robotics in the ICU were complex and dualistic. While there was excitement about the innovation and the potential efficiency gains, there was also skepticism about the practicality and readiness of such technologies for the sensitive ICU milieu. The prevailing belief among clinicians is that, while numerous tasks in the ICU could potentially be automated, the present state of robotic technology is best suited for delegating routine tasks to alleviate the heavy workload burden from the clinical staff.

### Participant Insights and Perceptions

Guided by insights from clinicians, our coding analysis revealed a total of 78 tasks, where robotic systems might be introduced into ICU practice (Multimedia Appendix 4). Of these tasks, 50 (64%) were primarily related to direct patient care (eg, repositioning patients and assisting with simple procedures), whereas 19 (24%) focused on indirect patient care activities such as delivering supplies or cleaning. An additional 6 (8%) tasks addressed administrative roles (eg, handling patient call lights), and 3 (4%) were classified as mixed, encompassing both direct and indirect elements (eg, sitting with a patient to help keep them calm). These tasks are systematically categorized according to primary codes from our coding analysis. Recognizing the intricate nature of ICU operations, we acknowledge that some tasks may be multifaceted, overlapping across multiple categories. For clarity and coherence, tasks that serve similar purposes have been consolidated and are detailed in Multimedia Appendix 5.

A notable finding from our analysis is the significant number of tasks identified under the category of “direct—performing procedure,” where 30 specific tasks were listed as shown in Multimedia Appendix 5. This category encapsulates tasks where robots could directly engage in patient care procedures, highlighting a substantial potential for robotic assistance in hands-on care activities. Additionally, the category “direct—supporting” includes 9 tasks, reflecting the supportive roles that robots could play in enhancing the efficiency and effectiveness of direct patient care.

**Figure 1:**
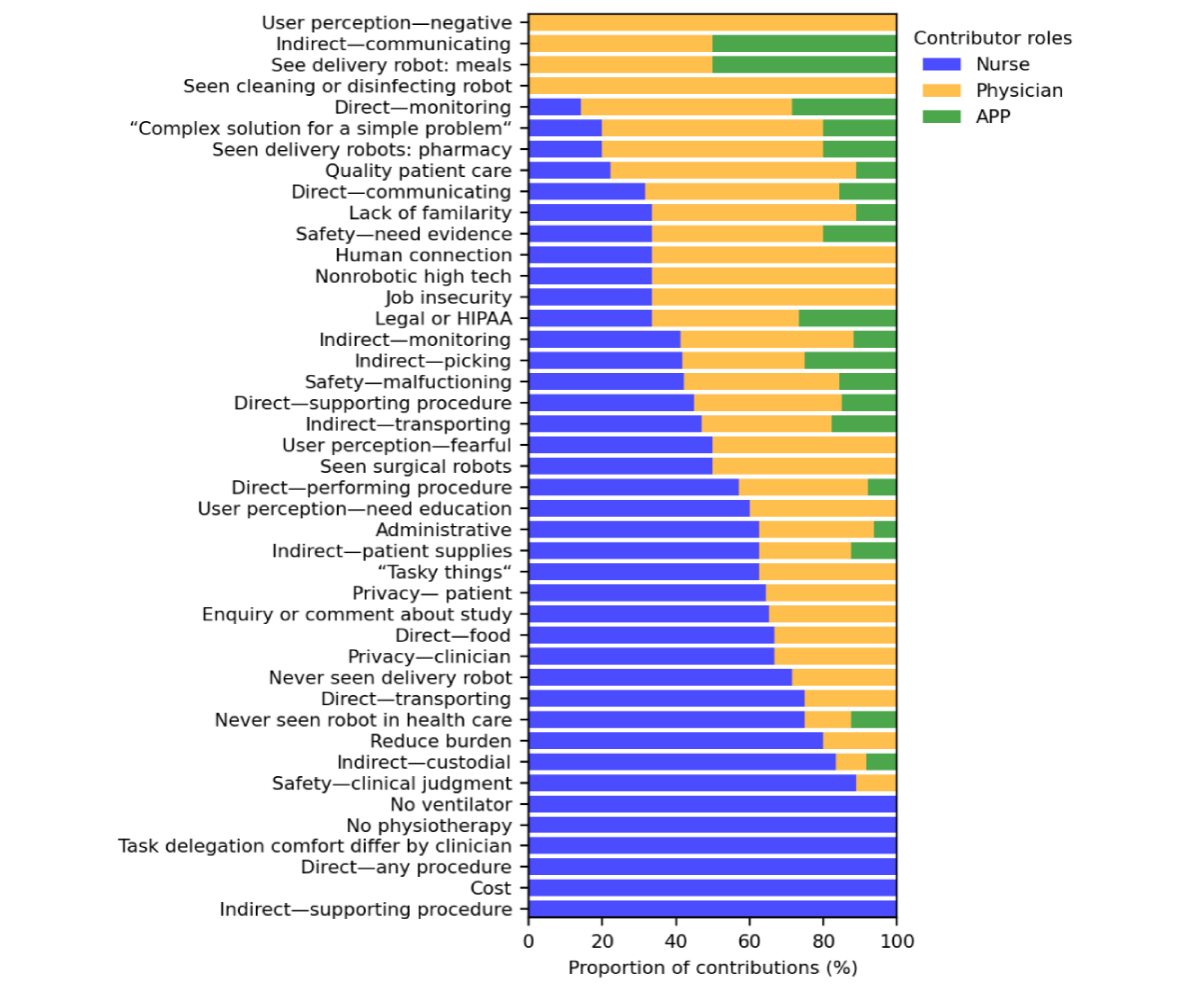
Distribution of thematic contributions by clinician role in a qualitative intensive care unit robotics study. This stacked bar chart illustrates the percentage of statements contributed by 7 nurses (blue), 6 physicians (orange), and 2 advanced practice providers (green) across distinct primary codes derived from interviews at 5 Southeastern United States hospitals between January and August 2023. Technical codes are removed. Each code on the horizontal axis represents a thematic category identified through directed content analysis, and the vertical axis shows the proportion of total statements. APP: advanced practice provider; HIPAA: Health Insurance Portability and Accountability Act.

The distribution of statements across various secondary codes, segmented by participant group (nurses, physicians, and APPs), is illustrated (Table 3). The table provides a quantitative overview of the engagement and contributions of each group in different thematic areas. The “nurse count,” “physician count,” and “APP count” columns represent the number of participants from each respective group who contributed to the discussions on each secondary code, while the “statements” columns reflect the total number of comments made by each group. In Figure 1, the stacked bar chart shows the proportion of statements contributed by nurses, physicians, and APPs for each primary code, providing a visual overview of how each group engaged with different thematic areas. Primary codes in “minimum capacity” were removed to maintain clarity and ensure emphasis on the most relevant thematic categories.

**Table 3:** Distribution of discussion topics across secondary codes among 15 intensive care unit clinicians.

Secondary codes	Participant count					Statements count			
	Total, n	Nurse, n (%)	Physician, n (%)	APP^a^, n (%)	Total, n		Nurse, n (%)	Physician, n (%)	APP, n (%)
Administrative	10	5 (50)	4 (40)	1 (10)	16		10 (63)	5 (31)	1 (6)
Comment or questions about the interview	10	6 (60)	4 (40)	0 (0)	23		15 (65)	8 (35)	0 (0)
Concerns	15	7 (47)	6 (40)	2 (13)	109		49 (45)	48 (44)	12 (11)
Demographic	15	7 (47)	6 (40)	2 (13)	30		15 (50)	12 (40)	3 (10)
Direct patient care	14	6 (43)	6 (43)	2 (14)	125		64 (51.2)	48 (38.4)	13 (10.4)
Indirect patient care	14	7 (50)	5 (36)	2 (14)	71		38 (54)	22 (31)	11 (15)
Minimum capacity	15	7 (47)	6 (40)	2 (13)	61		26 (43)	25 (41)	10 (16)
Not codable	3	2 (67)	0 (0)	1 (33)	5		2 (40)	0 (0)	3 (60)
Perceptions	11	5 (45)	5 (45)	1 (10)	32		19 (59)	12 (38)	1 (3)

^a^APP: advanced practice provider.

The “concerns” and “direct patient care” categories were the most frequently discussed topics, with nurses and physicians contributing the most statements, indicating shared apprehensions and interests. The “indirect patient care” and “minimum capacity” categories also received considerable attention from all participant groups, with a notably consistent number of statements from physicians and nurses. This suggests a common understanding of the importance of support tasks and the fundamental capabilities that are deemed necessary for robotic systems in the ICU.

Detailed distribution of statements across various primary codes is listed in Multimedia Appendix 6. The chi-square test of independence yielded a chi-square statistic of 112.87 with 102 degrees of freedom and a *P* value of .22. These results indicate no statistically significant differences in the distribution of thematic codes across nurses, physicians, and APPs, suggesting a relatively uniform contribution to the identified themes.

### Participant Evaluation of Proposed Tasks

**Figure 2:**
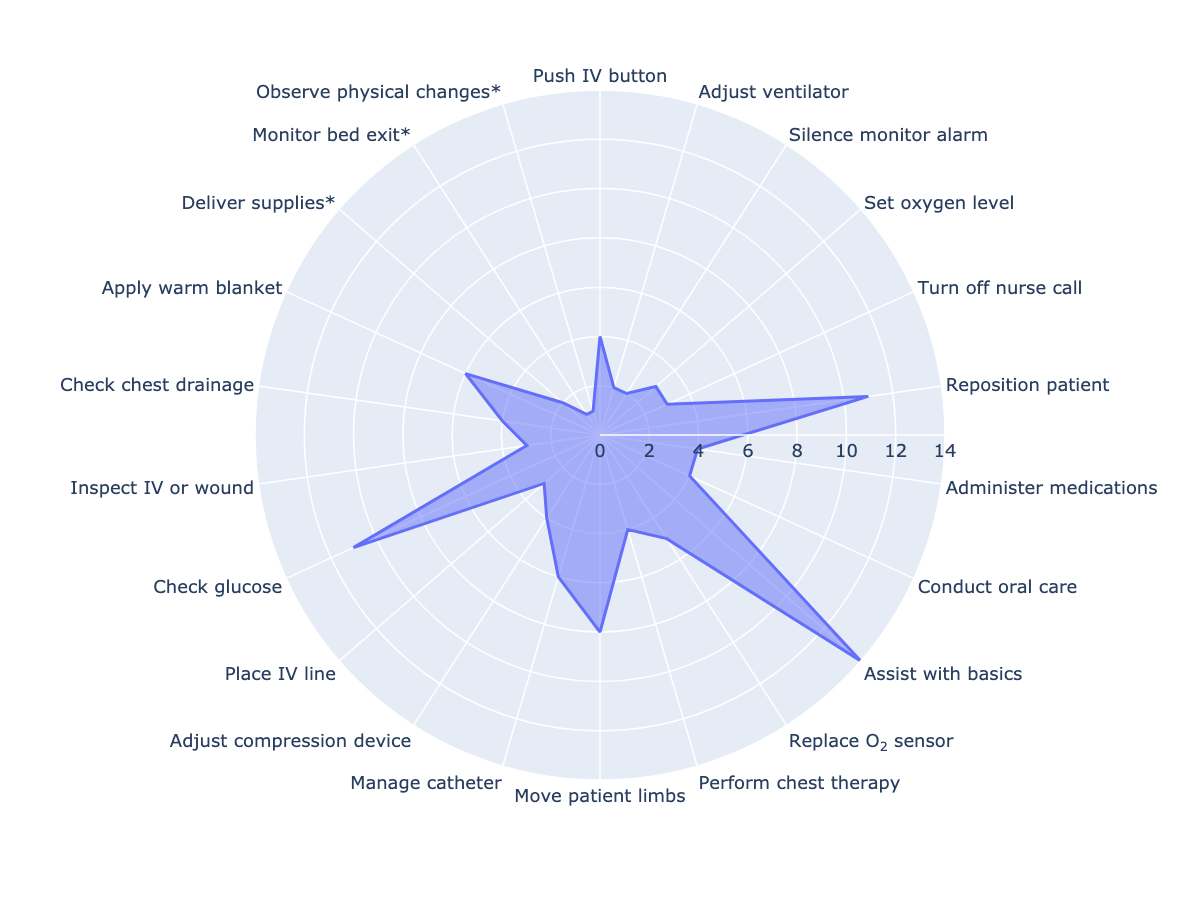
Radar chart of clinician-prioritized robotic tasks in intensive care unit settings. This radar chart illustrates the frequency with which 15 intensive care unit clinicians ranked each task within their top 7. Tasks involve a range of direct, indirect, and administrative care activities, with * highlighting newly suggested tasks. IV: intravenous.

**Figure 3:**
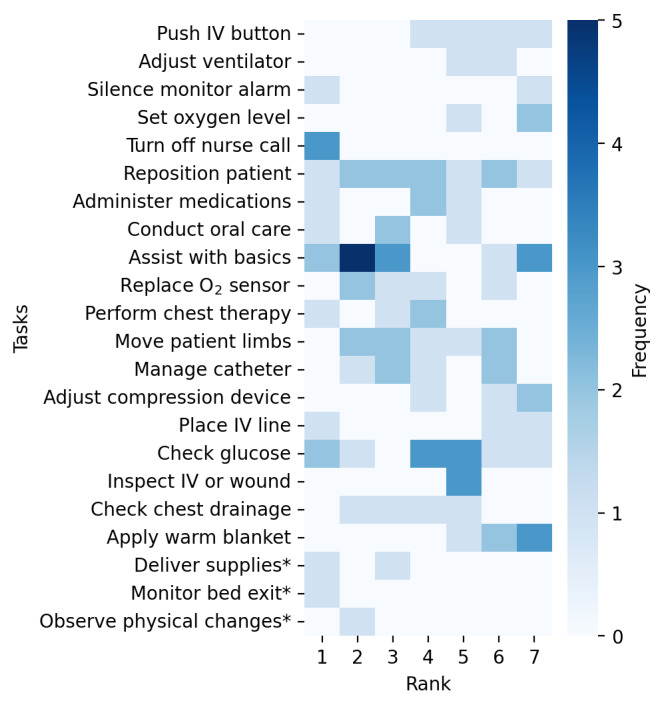
Heatmap of clinician-prioritized robotic tasks in intensive care unit settings. This heatmap shows task rankings by 15 intensive care unit clinicians (7 nurses, 6 physicians, and 2 advanced practice providers) from 5 Southeastern United States hospitals between January and August 2023. Darker cells indicate higher priority for robotic assistance, and tasks marked with * were newly proposed by participants. IV: intravenous.

Following the completion of the interviews, clinicians were presented with our preprepared list from the literature of 19 health care tasks performed by robots. There was some overlap with the tasks discussed during the interviews as well as previously unmentioned tasks. Participants were asked to rank the top 7 tasks they believe could be performed by a robot in the ICU. They could also add tasks not on the preprepared list. This yielded a total of 22 tasks for consideration. A radar and heatmap charts visualizing the rankings (Figures 2 and 3) demonstrate the variance in priority assigned to each task by the different clinician groups. The tasks are denoted by short codes for brevity, with detailed descriptions provided in Multimedia Appendix 3. These 2 figures succinctly illustrate the areas where clinicians feel robotic assistance would be most beneficial in the ICU, reflecting their assessment of where robots could best support the clinical team and patient care.

Interestingly, tasks perceived as less critical to direct clinical judgment were ranked higher. These tasks included controlling the nurse call button to mitigate interruptions, acting as a personal assistant for patient-related nonclinical tasks, and performing routine glucose checks. Conversely, tasks that require more nuanced clinical judgment, such as push button on the intravenous machine, adjusting ventilator, and adjust oxygen level from wall fixtures, while ranked, were not as highly prioritized, suggesting clinicians’ preference for retaining direct control over complex decision-making processes.

The radar chart reveals a pattern of prioritization among clinicians, with tasks such as acting as the personal assistant, performing glucose checks, and repositioning or boosting a patient in bed consistently appearing as top-ranked tasks. This indicates clinicians’ clear preference for assigning routine, recurring tasks requiring less clinical judgment to robotics. However, while these tasks are generally seen as routine, they may not always be suitable for autonomous robotic execution without human oversight. Tasks such as repositioning a patient can vary significantly in complexity depending on the patient’s condition. For patients with chest tubes, drains, pressure wounds, or those on ventilators, such movements require heightened situational awareness and clinical judgment.

### Survey Responses

**Figure 4:**
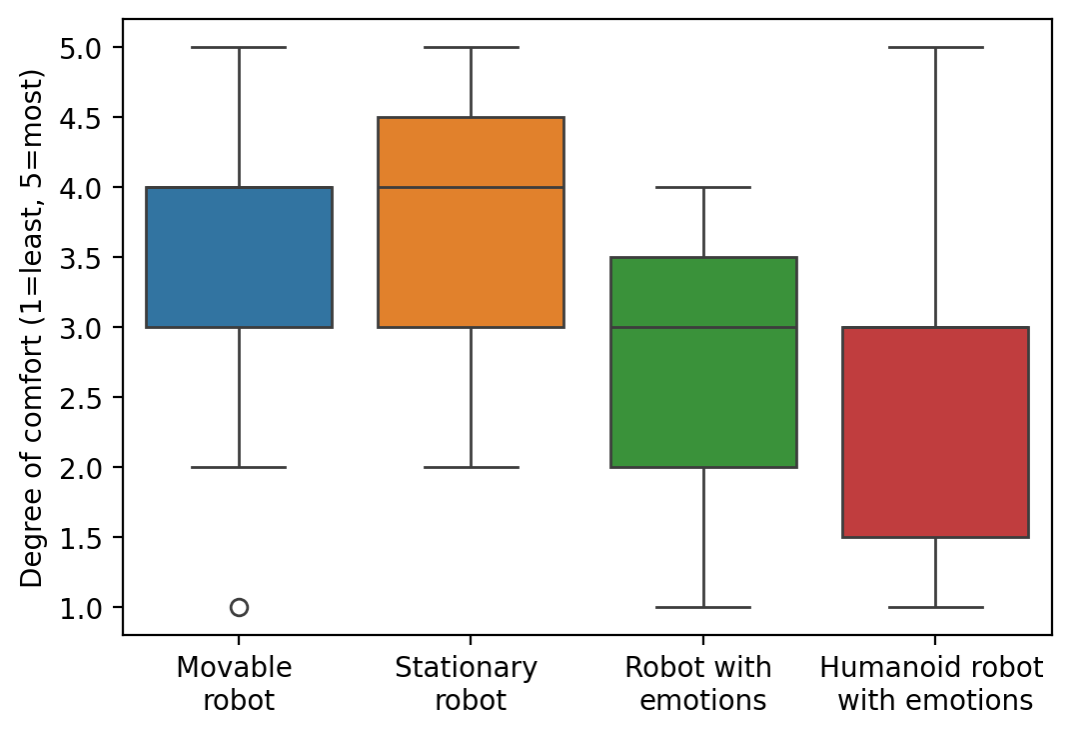
Box plot that compares clinicians’ comfort levels with different types of robots in the intensive care unit. This box plot compares self-reported comfort levels (1-5 scale) among 15 intensive care unit clinicians (7 nurses, 6 physicians, and 2 advanced practice providers) across 4 categories of robots—movable, stationary, a robot expressing emotions, and a humanoid robot with emotions. Data were collected between January and August 2023 from 5 Southeastern US hospitals. The vertical axis indicates the degree of comfort, and the horizontal axis lists each robot type, revealing varying levels of acceptance for different robotic designs and capabilities in high-acuity health care environments.

Figure 4 presents box plots of responses collected from the survey. There is a similar level of median comfort for both movable and stationary robots, indicating no significant preference for robot mobility. Additionally, the survey responses indicate a division in opinion regarding the simultaneous operation of multiple robots in the ICU, with a majority of 9 (60%) participants comfortable with the idea. In contrast, the remaining 6 (40%) participants expressed discomfort with multiple robots performing tasks concurrently.

Robots that can express emotions and humanoid robots are met with a lower median comfort level compared to the other types, which may reflect clinicians’ uncertainties or concerns about the unpredictability and implications of such features in a clinical setting.

## Discussion

### Principal Findings

Our study reveals a broad receptiveness among ICU clinicians to the integration of robotic technology, building on mounting evidence that such innovations can reduce staff burden and enhance care delivery in critical settings. While prior investigations have noted that telepresence and task-oriented robots can enhance workflow and reduce clinical workload, our qualitative analysis contributes a more nuanced view by detailing a diverse range of potential tasks—78 in total—where robots might assist without compromising patient safety or clinical judgment. This list offers valuable insights for future research and development, guiding the creation of technologies that align with the real-world needs and preferences of health care professionals.

### Clinician Preferences for Robotic Assistance

Clinicians prioritized tasks for robotic assistance that are routine, recurrent, and do not necessarily require clinical judgment. High-frequency, low-complexity tasks such as responding to the nurse call button and performing glucose checks were favored for robotic automation. This suggests that clinicians are interested in leveraging robotic technology to reduce the burden of basic tasks, potentially increasing efficiency and allowing them to focus on more patient-centric care.

On the other hand, tasks requiring higher clinical judgment, such as adjusting ventilator settings, were not as highly prioritized for robotic assistance. An illustrative comment from a nurse highlighted this: “One of the things nurses always learn is that you’re not treating the monitor. You’re treating a patient so there’s plenty of time that the monitor is wrong, and we have to use our nursing judgment.” Although such tasks are recognized as potentially automatable, clinicians expressed a need to maintain human oversight for these critical interventions, underlining an essential preference for direct human involvement in high-stakes decision-making and the delivery of compassionate care. This highlights a key insight that clinicians may prefer to retain control over critical tasks, possibly due to concerns about the consequences of errors and the current limitations of robotic technology in making nuanced decisions.

These insights have significant implications for the design and integration of robotic systems in the ICU. There is an opportunity for technology developers to focus on creating robotic systems that are seen as helpers rather than replacements for clinicians. Systems that can seamlessly integrate into the ICU environment and perform tasks that are perceived as helpful by clinicians are likely to be more readily accepted. Furthermore, the mixed comfort levels with humanoid robots and robots that express emotions suggest that while some clinicians might find these features enhance the patient experience, others might view them as unnecessary or potentially unsettling. This indicates a need for careful consideration of the human-robot interaction design and for the provision of adequate training and familiarization for clinicians.

### Ethical and Safety Considerations

Indirect references to the foundational biomedical ethical principles of autonomy, beneficence, nonmaleficence, and justice were reflected in many of the views shared by participants. With respect to autonomy, beneficence, and nonmaleficence, 10 participants explicitly stated that robots in patient rooms must comply with HIPAA (Health Insurance Portability and Accountability Act) protections regarding the privacy and security of patients’ individually identifiable personal health information. This is a requirement throughout the health care setting regardless of the introduction of robots. However, 5 participants believed that robots with audiovisual features would pose increased risks for breaches of patient privacy and confidentiality. In contrast, 3 participants noted that while patient personal health information must be protected, patients currently have decreased personal privacy in ICU by design (eg, glass doors and walls) to help clinicians monitor them for safety. Two participants asserted that monitoring for safety (regardless of machine or human) is reasonable if it improves response times and quality of patient care, provided that data are kept secure. One participant was concerned with the possibility of recordings “being used against staff.” Three other participants noted that if clinicians are practicing within scope and protocol, they should not worry about being recorded by the hospital. Two other participant comments around privacy and confidentiality were the use of robots in direct patient care should be stated on the consent to receive treatment form, and there should be indicators (eg, a light) when robots or other devices are using audiovisual features.

Specific to beneficence and nonmaleficence, all participants expressed concern about patient safety to varying degrees if robots perform direct patient care. Three participants described how ICU nurses use clinical judgment when treating patients who are critically ill. They described this as “treating the patient” and not responding to clinical device readings in isolation. They did not believe robots would be capable of applying this often experiential clinical decision-making. However, 3 other participants noted that errors and injuries currently occur in hospitals. Akin to safety concerns, 2 nurses explicitly stated that they would want 24×7 support in troubleshooting any robot malfunctions.

Related to justice, one participant noted their belief, “patients who might have limited health literacy or are older ... might be more likely to go to an ICU ... more likely to have complications for all sorts of reasons ... the people who would benefit most from technology tend to already have high health literacy [which leads to the concern of social and identity] inequity.” Further patients with a lower understanding about technology or who are in a compromised psychological state such as delirium might be frightened or confused by the presence of robots.

The focus on patient and clinician privacy and safety as well as the concern about missing the “human touch” underscore the need for patient, family, and staff education when deploying robots into the clinical setting to address these concerns. Resources or support would be needed for clinicians to detect and possibly troubleshoot issues with the robots. Three participants noted that their confidence in robots performing direct patient care would increase when they observed this occurring in their clinical setting without any complications. One further commented that robot functions in the ICU should be limited to functions posing less risk to patients and with patients themselves who are less medically fragile. Once the use of robots in ICUs becomes more proven and standardized, then functionality can be increased.

An ethics-related concern emerged about the loss of human connection when robots are included in direct care, regardless of whether process efficiencies are gained. Six clinicians made statements such as patients in ICU are “already prone to feeling isolation ... feeling like there wasn’t a person in there with them enough.” Would patients miss the human touch or feel “less than” with a robot caregiver? What impact would this have on patients and clinicians? A patient-centered approach to incorporating robots into the ICU setting would be to identify tasks, interactions, and possibly specific patients requiring the expression of human compassion and caring and preserve the human connection in those contexts [27,28].

### Strengths and Limitations

This study has several limitations. In addition to the small sample size, which restricts generalizability, there is also a possibility of selection bias, as clinicians who chose to participate may have had greater interest in or familiarity with robotics. Moreover, participants came from a limited geographic region, which may not reflect the diversity of ICU settings across different health care systems and patient populations. Since this study is cross-sectional, it only reflects clinicians’ perceptions at a single point in time, without accounting for how attitudes toward robotics may evolve as technology advances. Future investigations should include larger, more diverse samples from multiple institutions and regions, allowing researchers to draw broader conclusions. It would also be beneficial to conduct longitudinal studies to observe how the comfort levels and task preferences of clinicians shift over time with increasing exposure to robotic systems. Finally, collecting input from patients and their representatives is critical to fully understanding the ethical and practical ramifications of integrating robotics into patient care.

### Conclusions

This study aimed to explore the perceptions of ICU clinicians regarding the integration of robotics in critical care, focusing on identifying tasks where robots can effectively reduce clinician workload. Our findings confirm that clinicians view robotics as a supportive tool rather than a replacement for human expertise. By aiding with routine and physically demanding tasks, robots can free providers and nurses to focus on more complex, human-centric aspects of patient care. However, the ethical dimension—encompassing privacy, patient autonomy, and the preservation of the clinician-patient relationship—remains paramount. Ensuring robust oversight of robotics in the ICU, coupled with appropriate safeguards for patient data and clinician well-being, is essential for successful and responsible adoption.

## Appendix

Multimedia Appendix 1 Interview script for intensive care unit clinicians.

Multimedia Appendix 2 Google Forms questionnaire.

Multimedia Appendix 3 Collected list of robotic tasks from literature.

Multimedia Appendix 4 Unique tasks for robotic assistance in intensive care unit.

Multimedia Appendix 5 Categorized tasks for robotic assistance in intensive care unit.

Multimedia Appendix 6 Distribution of discussion topics among health care providers.

## Abbreviations

APP: advanced practice provider
HIPAA: Health Insurance Portability and Accountability Act
ICU: intensive care unit
IRB: institutional review board

## Data Availability

The datasets generated or analyzed during this study are not publicly available due to institutional privacy regulations and the sensitive nature of the data but are available from the corresponding author on reasonable request.
