# Supplementary material for: Clinicians’ Perceptions and Potential Applications of Robotics for Task Automation in Critical Care: Qualitative Study
Source: J Med Internet Res. 2025 Mar 28;27:e62957. doi: 10.2196/62957 (PMC11992484; doi:10.2196/62957)
Supplement: Multimedia Appendix 1 [file jmir_v27i1e62957_app1.docx]

Hi _____, nice to meet you. Thank you for participating in our study on ICU robotics. Is it ok if we record this meeting for our reference later?

To begin the interview session, we will start with some general questions for the first 30 minutes. For the rest of the interview, we will need you to fill out a google form and you will be asked to provide your email to receive the Amazon gift card.

Remember your compensation is independent of the response to any of the questions asked during the interview. This recording will be confidential and only authorized researchers can access. You are allowed to withdraw from this study at any time point.

***Opening Questions***

1. Please introduce yourselves with your name, title, and how long you have worked in ICU.

2. (We display robotics-type definitions and their related pictures.) Here we do not discuss surgical robots (DaVinci, etc.), which is a type of professional service robot. A collaborative robot is defined as one that is designed for direct interaction with humans within a defined collaborative workspace. A professional service robot is defined as a semi- or fully autonomous robot for the automation of commercial tasks, excluding manufacturing operations.

***Introductory Questions***

1. Can you identify or provide examples of robots that you have seen in the ICU or any part of the hospital? (Probe question: What about the mail robot in the hospital?) Can you briefly say what they do? Or what do you think they do? (If no, say “That is fine, let’s proceed to the next question)

***Transition Questions***

1. What are some of the tasks that can be performed by the robot in the ICU environment without direct patient contact? (Probe question: What are some of the observations that you monitor on patients that have already been captured by the vital signs machines or other machines in the ICU?)

2. What are some of the tasks that can be performed by the robot in the ICU environment involving direct patient contact? (Probe question: What are some tasks you do over and over again that are standard?)

***Key Questions***

1. What is the minimum capacity/ functionality that an ICU robot should be able to perform? (Probe question: What about cameras, audio capture, and wheels?)

2. What concerns do you have about robots co-assisting clinical care in the ICU? (Probe question: Would you have any concerns about the camera or recording devices?)

***Ending Questions***

For the rest of the interview, we will need you to fill out a google form. If you have any questions while filling out the google form, feel free to ask us. When you are filling out the form, you will see a list of tasks (Appendix II) and feel free to come up with new tasks or put in the tasks you mentioned before. Do you have additional comments or questions? Lead into survey session.
