# Supplementary material for: Clinicians’ Perceptions and Potential Applications of Robotics for Task Automation in Critical Care: Qualitative Study
Source: J Med Internet Res. 2025 Mar 28;27:e62957. doi: 10.2196/62957 (PMC11992484; doi:10.2196/62957)
Supplement: Multimedia Appendix 2 [file jmir_v27i1e62957_app2.docx]

1. What is your name? (Response: Free text)

2. What is your age? (Response: Free text)

3. What is your gender/ identity? (Response: Free text)

4. What is your position in the ICU department? (Response: Free text)

5. How long have you been working in the ICU? If you have other experiences in the healthcare field before, please state them. (Response: Free text)

6. Please review this collected list of robotic tasks and answer the following questions (You can also include any additional tasks that might be useful including the tasks you might have mentioned during focus group study):

- Please rank the #1 task in the above list that you want an ICU robot to perform or assist performing. (Response: Rank order)
- Please rank the #2 task in the above list that you want an ICU robot to perform or assist performing. (Response: Rank order)
- Please rank the #3 task in the above list that you want an ICU robot to perform or assist performing. (Response: Rank order)
- Please rank the #4 task in the above list that you want an ICU robot to perform or assist performing. (Response: Rank order)
- Please rank the #5 task in the above list that you want an ICU robot to perform or assist performing. (Response: Rank order)
- Please rank the #6 task in the above list that you want an ICU robot to perform or assist performing. (Response: Rank order)
- Please rank the #7 task in the above list that you want an ICU robot to perform or assist performing. (Response: Rank order)
- Please provide your reasons for ranking the above list. (Optional) (Response: Free text)

7. What degree of comfort do you have with a movable robot (Robot with wheels) in the ICU? (Response: Likert scale)

8. What degree of comfort do you have with a stationary robot (Robot mounting on the bed or stand on the floor) in the ICU? (Response: Likert scale)

9. Do you believe that robotic systems that exhibit human emotions may improve/ disrupt interactions with care providers and/or families? (Response: Likert scale)

10. What degree of comfort do you have with a humanoid robot that exhibits emotions in the ICU? (Response: Likert scale)

11. In an ideal scenario, would you consider multiple robots performing tasks simultaneously? (Response: Yes/ No choice)

12. How have your perceptions of robotics in the ICU changed from the beginning of this focus group study? (Response: Likert scale)

13. Is there anything else you would like to share about your feelings or beliefs about using robots in the ICU environment? (Response: Free text)
