# Supplementary material for: Clinicians’ Perceptions and Potential Applications of Robotics for Task Automation in Critical Care: Qualitative Study
Source: J Med Internet Res. 2025 Mar 28;27:e62957. doi: 10.2196/62957 (PMC11992484; doi:10.2196/62957)
Supplement: Multimedia Appendix 3 [file jmir_v27i1e62957_app3.docx]

| **Number** | **Task Short Code** | **Detailed Task** |
| --- | --- | --- |
| 1 | Push IV button | Push a button on the intravenous (IV) pole machine. |
| 2 | Adjust ventilator | Adjust a ventilator knob used for critically ill machine-ventilated patients. |
| 3 | Silence monitor alarm | Push a button on an ICU monitor to “Silence alarm” false alerts. |
| 4 | Set oxygen level | Adjust the level of oxygen (L/min) from wall fixtures. |
| 5 | Turn off nurse call | Push the nurse call button to “Off” on the wall. |
| 6 | Reposition patient | Reposition or boost a patient in bed. |
| 7 | Administer medications | Bring meds in a med cup to the patient to administer. |
| 8 | Conduct oral care | Conduct oral care for patients with COVID-19 who are too weak to do this care. |
| 9 | Assist with basics | Act as the personal assistant, helping patients with basic tasks that they usually ask their families to do (eg, lights on and off, TV channel change). |
| 10 | Replace O2 sensor | Replace oxygen saturation sensor on the fingertip. |
| 11 | Perform chest therapy | Conduct chest physiotherapy. |
| 12 | Move patient limbs | Do range of motion (basic or passive) activities with patient. |
| 13 | Manage catheter | Tip or empty the Foley catheter and empty the urinal. |
| 14 | Adjust compression device | Help take sequential compression device on or off. |
| 15 | Place IV line | Place a peripheral IV line. |
| 16 | Check glucose | Perform glucose checks, especially for patients receiving an insulin drip. |
| 17 | Inspect IV/wound | Visualize an IV site or a wound. |
| 18 | Check chest drainage | Visualize chest tube output in the chest drainage system (Pleur-evac; Teleflex Inc). |
| 19 | Apply warm blanket | Apply a warm blanket. |
| **Additional tasks proposed by participants** | | |
| 20 | Deliver supplies | Supply delivery. |
| 21 | Monitor bed exit | Monitor patient for getting out of bed unassisted. |
| 22 | Observe physical changes | Monitor patients’ physical changes (skin color changed, respiratory rate, sweating. etc). |
