# Supplementary material for: Clinicians’ Perceptions and Potential Applications of Robotics for Task Automation in Critical Care: Qualitative Study
Source: J Med Internet Res. 2025 Mar 28;27:e62957. doi: 10.2196/62957 (PMC11992484; doi:10.2196/62957)
Supplement: Multimedia Appendix 4 [file jmir_v27i1e62957_app4.docx]

| **Task** | **Specific Tasks** | **Primary Codes** |
| --- | --- | --- |
| 1 | “Correlate” with ventilator settings | Direct - Communicating Direct - Monitoring Direct - Performing Procedure |
| 2 | Accompany rounding teams to display imaging & labs | Administrative |
| 3 | Administrate blood | Direct - Supporting Procedure |
| 4 | Administrate medication | Direct - Performing Procedure |
| 5 | Alert clinicians in a direct way, not just alarm beeps | Indirect - Communicating Indirect - Monitoring |
| 6 | Answer call lights | Administrative |
| 7 | Answer phone calls | Administrative |
| 8 | Apply warm/ cooling blankets for patients | Direct - Performing Procedure |
| 9 | Ask the patient post-op pain scores | Direct - Communicating Direct - Monitoring |
| 10 | Assist patients with physiotherapy | Direct - Performing Procedure |
| 11 | Assist patients with range of motion exercises | Direct - Performing Procedure |
| 12 | Assist prompting for incentive spirometry | Direct - Communicating |
| 13 | Assist with feeding | Direct - Food |
| 14 | Assist with lifting | Direct - Supporting Procedure |
| 15 | Assist with patients ambulation requiring more than 1 clinician (e.g., to manage IV poles, to steady/support the patient, etc.) | Direct - Supporting Procedure |
| 16 | Bathe the patient (e.g., CHG bath) | Direct - Performing Procedure |
| 17 | Carry monitor when staff ambulates patients | Direct - Supporting Procedure |
| 18 | Change linen (bags) | Indirect - Custodial |
| 19 | Check cognition (e.g., Hourly neurochecks) | Direct - Communicating Direct - Monitoring |
| 20 | Check devices and lines to ensure functionality | Direct - Performing Procedure |
| 21 | Check serum glucose (blood sugar) - esp. 2 hour/frequent checks | Direct - Performing Procedure |
| 22 | Clean and vacuum floors | Indirect - Custodial |
| 23 | Clean the patient (stool, urine, vomit, bodily fluids) | Direct - Performing Procedure |
| 24 | Collect patient data | Administrative |
| 25 | Communicate with providers | Indirect - Monitoring |
| 26 | Conduct CPR compressions | Direct - Performing Procedure |
| 27 | Control lights and remote | Indirect - Supporting Procedure |
| 28 | Count CPR rounds and alert when to check for pulse or give another medication | Direct - Supporting Procedure |
| 29 | De-line the patient | Direct - Performing Procedure |
| 30 | Deliver blood | Indirect - Transporting |
| 31 | Deliver meal | Direct - Food |
| 32 | Deliver medications | Indirect - Transporting |
| 33 | Discharge the patient with wheelchair | Direct - Transporting |
| 34 | Document patient charts and follow care providers | Administrative |
| 35 | Draw blood for labs | Direct - Performing Procedure |
| 36 | Empty Foley catheter | Direct - Performing Procedure |
| 37 | Foster family comfort | Direct - Communicating |
| 38 | Hold the patient | Direct - Performing Procedure |
| 39 | Hold/Pass instruments (e.g., Central and arterial line placement, IV pole) | Direct - Supporting Procedure |
| 40 | Import vital signs from monitor | Indirect - Monitoring |
| 41 | Manage drips | Direct - Performing Procedure |
| 42 | Manage patients flow (e.g., Room assignments & Alert when rooms are ready) | Administrative |
| 43 | Measure urine output | Direct - Performing Procedure |
| 44 | Monitor patient and prevent fall | Direct - Communicating Indirect - Monitoring |
| 45 | Monitor vital signs & alert providers | Indirect - Monitoring |
| 46 | Move the patient (from one place to another place) | Direct - Performing Procedure |
| 47 | Order meals | Direct - Communicating |
| 48 | Perform one-on-one observations for patients (e.g. Delirium, substance withdrawal) | Indirect - Monitoring Direct - Communicating |
| 49 | Pick and deliver medications | Indirect - Picking Supplies |
| 50 | Pick and retrieve supplies (e.g. linens, warm blankets, extra pillows, central lines, wound care) | Indirect - Picking Supplies |
| 51 | Placing IVs | Direct - Performing Procedure |
| 52 | Prompt patients to move within their bed | Direct - Communicating |
| 53 | Push buttons to adjust medications based on programmed parameters | Direct - Performing Procedure |
| 54 | Put on TED hose (compression stockings) | Direct - Performing Procedure |
| 55 | Read lab results and make suggestions to change ventilator settings | Indirect - Communicating |
| 56 | Reposition the bed | Direct - Performing Procedure |
| 57 | Restock supplies | Indirect - Custodial |
| 58 | Retrieve supplies (e.g. linens, warm blankets, extra pillows, central lines, wound care) | Indirect - Delivering Supplies Indirect - Transporting |
| 59 | Set up blood transfusions | Direct - Performing Procedure |
| 60 | Set up equipment (e.g., CRRT) | Direct - Supporting Procedure |
| 61 | Set up meal trays | Direct - Food |
| 62 | Set up room prior to patient arrival | Indirect - Custodial |
| 63 | Set up the equipment to turn larger patients (e.g., Hoyer lifts) | Direct - Supporting Procedure |
| 64 | Set up tube feeding | Direct - Performing Procedure |
| 65 | Silence the false alarm | Indirect - Supporting Procedure |
| 66 | Sit with patients and keep them calm | Direct - Communicating Indirect - Monitoring |
| 67 | Start pre-programmed IV pumps | Direct - Performing Procedure |
| 68 | Take out trash | Indirect - Custodial |
| 69 | Test ventilator (e.g., Check tube position and ventilator parameters in certain modes) | Direct - Performing Procedure |
| 70 | Titrate medications | Direct - Performing Procedure |
| 71 | Transfer the patient from/ to bed to/ from chair or stretcher | Direct - Performing Procedure |
| 72 | Transfer the patient to bedside commode | Direct - Performing Procedure |
| 73 | Transport empty beds to the operation room | Indirect - Transporting |
| 74 | Transport lower acuity patients | Direct - Transporting |
| 75 | Transport the patient to the CT or MRI | Direct - Transporting |
| 76 | Transport ultrasound machine | Indirect - Transporting |
| 77 | Turn and reposition the patient | Direct - Performing Procedure |
| 78 | Wound care (e.g., change a dressing) | Direct - Performing Procedure |
