# Supplementary material for: Clinicians’ Perceptions and Potential Applications of Robotics for Task Automation in Critical Care: Qualitative Study
Source: J Med Internet Res. 2025 Mar 28;27:e62957. doi: 10.2196/62957 (PMC11992484; doi:10.2196/62957)
Supplement: Multimedia Appendix 5 [file jmir_v27i1e62957_app5.docx]

| **Primary Codes** | **Specific Tasks** |
| --- | --- |
| Administrative | - Answer call lights - Answer phone calls - Manage patients flow (e.g., Room assignments & Alert when rooms are ready) - Document patient charts and follow care providers - Accompany rounding teams to display imaging & labs - Collect patient data |
| Direct - Communicating | - Sit with patients and keep them calm - Perform one-on-one observations for patients (e.g., Delirium, Substance withdrawal) - Monitor patient and prevent fall - Ask the patient post-op pain scores - Prompt patients to move within their bed - Foster family comfort - “Correlate” with ventilator settings - Check cognition (e.g., Hourly neurochecks) - Order meals - Assist prompting for incentive spirometry |
| Direct - Food | - Assist with feeding - Deliver meal - Set up meal trays |
| Direct - Monitoring | - Ask the patient post-op pain scores - “Correlate” with ventilator settings - Check cognition (e.g., Hourly neurochecks) |
| Direct - Performing Procedure | - Transfer the patient to bedside commode - Turn and reposition the patient - Reposition the bed - Transfer the patient from/ to bed to/ from chair or stretcher - Move the patient (from one place to another place) - Hold the patient - De-line the patient - Clean the patient (stool, urine, vomit, bodily fluids) - Bathe the patient (e.g., CHG bath) - Apply warm/ cooling blankets for patients - Assist patients with range of motion exercises - Assist patients with physiotherapy - Set up tube feeding - Administrate medication - Titrate medications - Start pre-programmed IV pumps - Push buttons to adjust medications based on programmed parameters - Manage drips - Check devices and lines to ensure functionality - Placing IVs - Check serum glucose (blood sugar) - esp. 2 hour/frequent checks - Wound care (e.g., change a dressing) - Conduct CPR compressions - Put on TED hose (compression stockings) - “Correlate” with ventilator settings - Test ventilator (e.g., Check tube position and ventilator parameters in certain modes) - Set up blood transfusions - Draw blood for labs - Empty Foley catheter - Measure urine output |
| Direct - Supporting Procedure | - Hold/Pass instruments (e.g., Central and arterial line placement, IV pole) - Carry monitor when staff ambulates patients - Count CPR rounds and alert when to check for pulse or give another medication - Assist with patients ambulation requiring more than 1 clinician (e.g., to manage IV poles, to steady/support the patient, etc.) - Turn and reposition patients - Set up equipment (e.g., CRRT) - Set up the equipment to turn larger patients (e.g., Hoyer lifts) - Assist with lifting - Administrate blood |
| Direct - Transporting | - Transport the patient to the CT or MRI - Transport lower acuity patients - Discharge the patient with wheelchair |
| Indirect - Communicating | - Read lab results and make suggestions to change ventilator settings - Alert clinicians in a direct way, not just alarm beeps |
| Indirect - Custodial | - Take out trash - Change linen (bags) - Clean and vacuum floors - Restock supplies - Set up room prior to patient arrival |
| Indirect - Monitoring | - Import vital signs from monitor - Monitor vital signs & alert providers - Sit with patients and keep them calm - Perform one-on-one observations for patients (e.g. Delirium, substance withdrawal) - Monitor patient and prevent fall - Communicate with providers - Alert clinicians in a direct way, not just alarm beeps |
| Indirect - Delivering Supplies | - Retrieve supplies (e.g. linens, warm blankets, extra pillows, central lines, wound care) |
| Indirect - Picking Supplies | - Pick and retrieve supplies (e.g. linens, warm blankets, extra pillows, central lines, wound care) - Pick and deliver medications |
| Indirect - Supporting Procedure | - Control lights and remote - Silence the false alarm |
| Indirect - Transporting | - Transport empty beds to the operation room - Transport ultrasound machine - Retrieve supplies (e.g. linens, warm blankets, extra pillows, central lines, wound care) - Deliver blood - Deliver medications |
