# Supplementary material for: Clinicians’ Perceptions and Potential Applications of Robotics for Task Automation in Critical Care: Qualitative Study
Source: J Med Internet Res. 2025 Mar 28;27:e62957. doi: 10.2196/62957 (PMC11992484; doi:10.2196/62957)
Supplement: Multimedia Appendix 6 [file jmir_v27i1e62957_app6.docx]

|  | Participant count | | | | Statements Count | | | |
| --- | --- | --- | --- | --- | --- | --- | --- | --- |
| Primary Codes | **Total** | **Nurse** | **Physician** | **App** | **Total** | **Nurse** | **Physician** | **App** |
| "Complex solution for a simple problem" | 4 | 1 | 2 | 1 | 5 | 1 | 3 | 1 |
| "Tasky things" | 6 | 3 | 3 | 0 | 8 | 5 | 3 | 0 |
| Administrative | 10 | 5 | 4 | 1 | 16 | 10 | 5 | 1 |
| Audio | 11 | 4 | 5 | 2 | 13 | 5 | 6 | 2 |
| Cost | 1 | 1 | 0 | 0 | 1 | 1 | 0 | 0 |
| Device Compatibility | 2 | 1 | 1 | 0 | 2 | 1 | 1 | 0 |
| Dexterity | 5 | 2 | 3 | 0 | 7 | 2 | 5 | 0 |
| Direct - Any Procedure | 1 | 1 | 0 | 0 | 1 | 1 | 0 | 0 |
| Direct - Communicating | 8 | 2 | 4 | 2 | 19 | 6 | 10 | 3 |
| Direct - Food | 6 | 4 | 2 | 0 | 9 | 6 | 3 | 0 |
| Direct - Monitoring | 7 | 1 | 4 | 2 | 7 | 1 | 4 | 2 |
| Direct - Performing Procedure | 13 | 5 | 6 | 2 | 63 | 36 | 22 | 5 |
| Direct - Supporting Procedure | 13 | 5 | 6 | 2 | 20 | 9 | 8 | 3 |
| Direct - Transporting | 3 | 2 | 1 | 0 | 4 | 3 | 1 | 0 |
| Enquiry/ Comment about study | 10 | 6 | 4 | 0 | 23 | 15 | 8 | 0 |
| Human Connection | 6 | 2 | 4 | 0 | 9 | 3 | 6 | 0 |
| Indirect - Communicating | 2 | 0 | 1 | 1 | 2 | 0 | 1 | 1 |
| Indirect - Custodial | 6 | 4 | 1 | 1 | 12 | 10 | 1 | 1 |
| Indirect - Monitoring | 7 | 3 | 3 | 1 | 17 | 7 | 8 | 2 |
| Indirect - Delivering Supplies | 6 | 4 | 1 | 1 | 8 | 5 | 2 | 1 |
| Indirect - Picking Supplies | 8 | 3 | 3 | 2 | 12 | 5 | 4 | 3 |
| Indirect - Supporting Procedure | 2 | 2 | 0 | 0 | 3 | 3 | 0 | 0 |
| Indirect - Transporting | 9 | 3 | 4 | 2 | 17 | 8 | 6 | 3 |
| Job Insecurity | 3 | 1 | 2 | 0 | 3 | 1 | 2 | 0 |
| Lack Of Familiarity | 7 | 2 | 4 | 1 | 9 | 3 | 5 | 1 |
| Legal/ HIPAA | 9 | 3 | 4 | 2 | 15 | 5 | 6 | 4 |
| Movement | 14 | 6 | 6 | 2 | 20 | 9 | 7 | 4 |
| No Physiotherapy | 1 | 1 | 0 | 0 | 1 | 1 | 0 | 0 |
| No Ventilator | 1 | 1 | 0 | 0 | 1 | 1 | 0 | 0 |
| Privacy - Patient | 9 | 5 | 4 | 0 | 14 | 9 | 5 | 0 |
| Privacy - Clinician | 6 | 4 | 2 | 0 | 6 | 4 | 2 | 0 |
| Quality Patient Care | 8 | 2 | 5 | 1 | 9 | 2 | 6 | 1 |
| Reduce Burden | 5 | 3 | 2 | 0 | 10 | 8 | 2 | 0 |
| Safety - Clinical Judgement | 5 | 4 | 1 | 0 | 9 | 8 | 1 | 0 |
| Safety - Malfunctioning | 12 | 5 | 5 | 2 | 19 | 8 | 8 | 3 |
| Safety - Need Evidence | 12 | 5 | 5 | 2 | 15 | 5 | 7 | 3 |
| Slam | 1 | 1 | 0 | 0 | 1 | 1 | 0 | 0 |
| Storage | 1 | 0 | 0 | 1 | 1 | 0 | 0 | 1 |
| Task delegation comfort differ by clinician | 1 | 1 | 0 | 0 | 1 | 1 | 0 | 0 |
| Touch Screen | 1 | 1 | 0 | 0 | 1 | 1 | 0 | 0 |
| Not Codable | 3 | 2 | 0 | 1 | 5 | 2 | 0 | 3 |
| User Perception - fearful (patients and clinicians) | 2 | 1 | 1 | 0 | 2 | 1 | 1 | 0 |
| User Perception – need education (patients and clinicians) | 4 | 2 | 2 | 0 | 5 | 3 | 2 | 0 |
| User Perception – negative (clinicians) | 1 | 0 | 1 | 0 | 1 | 0 | 1 | 0 |
| Video | 14 | 7 | 5 | 2 | 16 | 7 | 6 | 3 |
